# An Incomplete TCA Cycle Increases Survival of *Salmonella* Typhimurium during Infection of Resting and Activated Murine Macrophages

**DOI:** 10.1371/journal.pone.0013871

**Published:** 2010-11-08

**Authors:** Steven D. Bowden, Vinoy K. Ramachandran, Gitte M. Knudsen, Jay C. D. Hinton, Arthur Thompson

**Affiliations:** 1 Institute of Food Research, Norwich, United Kingdom; 2 Moyne Institute of Preventive Medicine, School of Genetics and Microbiology, Trinity College, Dublin, Ireland; University of Birmingham, United Kingdom

## Abstract

**Background:**

In comparison to the comprehensive analyses performed on virulence gene expression, regulation and action, the intracellular metabolism of *Salmonella* during infection is a relatively under-studied area. We investigated the role of the tricarboxylic acid (TCA) cycle in the intracellular replication of *Salmonella* Typhimurium in resting and activated macrophages, epithelial cells, and during infection of mice.

**Methodology/Principal Findings:**

We constructed deletion mutations of 5 TCA cycle genes in *S*. Typhimurium including *gltA, mdh*, *sdhCDAB, sucAB*, and *sucCD.* We found that the mutants exhibited increased net intracellular replication in resting and activated murine macrophages compared to the wild-type. In contrast, an epithelial cell infection model showed that the *S*. Typhimurium Δ*sucCD* and Δ*gltA* strains had reduced net intracellular replication compared to the wild-type. The glyoxylate shunt was not responsible for the net increased replication of the TCA cycle mutants within resting macrophages. We also confirmed that, in a murine infection model, the *S*. Typhimurium Δ*sucAB* and Δ*sucCD* strains are attenuated for virulence.

**Conclusions/Significance:**

Our results suggest that disruption of the TCA cycle increases the ability of *S*. Typhimurium to survive within resting and activated murine macrophages. In contrast, epithelial cells are non-phagocytic cells and unlike macrophages cannot mount an oxidative and nitrosative defence response against pathogens; our results show that in HeLa cells the *S*. Typhimurium TCA cycle mutant strains show reduced or no change in intracellular levels compared to the wild-type [1]. The attenuation of the *S*. Typhimurium Δ*sucAB* and Δ*sucCD* mutants in mice, compared to their increased net intracellular replication in resting and activated macrophages suggest that *Salmonella* may encounter environments within the host where a complete TCA cycle is advantageous.

## Introduction

*Salmonella enterica* is one of the most common food-borne bacterial pathogens and the disease outcomes range from a self-limited gastroenteritis to typhoid fever in mammals. Typhoidal *Salmonella* serovars, such as *Salmonella enterica* serovars Typhi and Paratyphi, cause an estimated 20 million cases of typhoid and 200,000 human deaths wordwide per annum [2]. Typhoid infection involves transmission of *Salmonella* via the ingestion of contaminated food and water followed by bacterial penetration of the small intestinal barrier by invading gut epithelial cells causing bloody diarrhoea. Subsequently, *Salmonella* can enter the mesenteric lymph nodes and invade phagocytic cells such as macrophages [3], [4]. Within macrophages, the *Salmonella* bacteria are compartmentalised into a modified intracellular phagosome termed the “*Salmonella* containing vacuole” (SCV). The SCV protects the *Salmonella* by preventing lysosomal fusion [5], [6]. The antimicrobial defences deployed by macrophages include reactive oxygen and reactive nitrosative intermediates (ROI and RNI respectively), as well as antimicrobial peptides [7], [8]. The ROI response is bactericidal and occurs approximately 1 h post-infection of macrophages whereas the RNI response is bacteriostatic and occurs approximately 8 h post-infection [9], [10], [11].

Recently, research is being directed towards establishing the role of central metabolic pathways in the virulence of pathogenic bacteria [12]. For example, it has been shown that fitness of *Escherichia coli* during urinary tract infection is reliant upon gluconeogenesis and the TCA cycle [13]. In *Salmonella*, we and others have demonstrated that glycolysis and glucose are required for the intracellular replication of *S*. Typhimurium in macrophages and mice [14], [15], [16]. Other work has shown that full virulence of *S*. Typhimurium strain SR11 in a murine infection model requires a complete TCA cycle [17].

**Figure 1:**
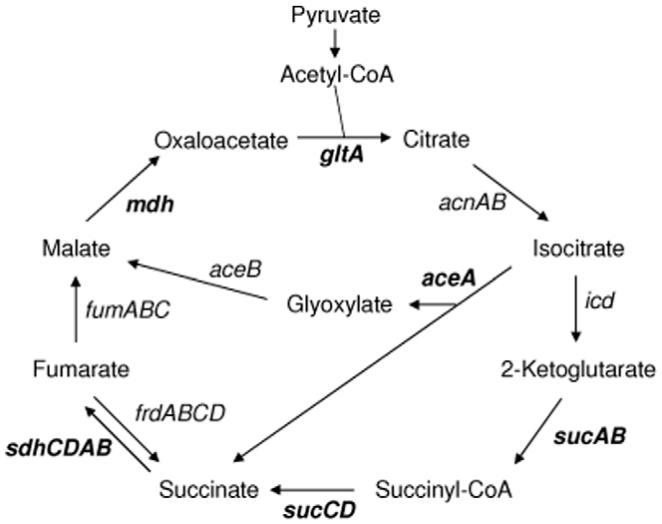
TCA cycle and glyoxylate shunt showing intermediate products and genes encoding enzymes within the pathway. Deleted genes are shown in bold.

In the current study, we investigated the effect of disrupting the TCA cycle on the ability of *S*. Typhimurium to replicate within murine RAW macrophages and HeLa epithelial cells by deleting genes encoding specific TCA cycle enzymes. The HeLa epithelial cell line is a well-defined model for infection of mammalian cells with *S.* Typhimurium, and has been used to characterize the biogenesis and evolution of the SCV [18], [19], [20]. We deleted the *sucAB*, *sucCD*, *sdhCDAB*, *mdh* and *gltA* genes which encode 2-ketoglutarate dehydrogenase, succinyl-CoA synthetase, succinate dehydrogenase, malate dehdrogenase and citrate synthase respectively (Figure 1) [21]. Interestingly, we found that disruption of the TCA cycle can result in an increase in the levels of the *S*. Typhimurium mutant strains within resting and activated macrophages compared to the wild-type. Mutants that showed increased net replication in macrophages did not exhibit the same phenotype in epithelial cells and, in agreement with previously published results, were severely attenuated in mouse infection assays compared to the wild-type [17].

## Materials and Methods

### Bacterial strains, growth conditions and reagents

*S*. Typhimurium strains and plasmids used in this work are listed in Table S1. All mutants were constructed in the wild-type strain 4/74 which is the prototrophic parent of the well-characterized SL1344 strain [22]. Strains were maintained in Luria-Bertani (LB) broth or on plates with appropriate antibiotics at the following concentrations; ampicillin (Ap, Sigma Aldrich), 50 µg.ml^−1^, chloramphenicol (Cm, Sigma Aldrich), 12.5 µg.ml^−1^ and kanamycin (Kn, Sigma Aldrich), 50 µg.ml^−1^. Oligonucleotide primers were purchased from Sigma Genosys or Illumina, (California).

### Mutant construction

*S*. Typhimurium mutant strains were constructed according to published procedures (Datsenko and Wanner 2000) and as briefly described in [14]. Primers used to construct the *S*. Typhimurium deletion mutant strains were purchased from Sigma-Genosys (Table 1). P22-derived transductants were screened on green agar plates to obtain lysogen-free colonies and all deletion mutants were transduced into a clean wild-type background [24]. The complete absence of the structural genes was verified by DNA sequencing of the deleted regions of the chromosome.

**Table 1:** Primers used to construct *S*. Typhimurium gene deletion mutants.

Primer Name	Primer DNA sequence
aceAredf	CTGGCTTAATTCACCACATAACAATATGGAGCATCTGCACGTGTAGGCTGGAGCTGCTTC
aceAredr	TATCTGTAGGCCCGGTAAGCGCAGCGCCACCGGGCATCAACATATGAATATCCTCCTTAG
gltAredf	TCCGGCAGTCTTAAGCAATAAGGCGCTAAGGAGACCGTAAGTGTAGGCTGGAGCTGCTTC
gltAredr	GTACCGGATGGCGAGGGTTGCGTCGCCATCCGGTTGTCAACATATGAATATCCTCCTTAG
icdAredf	TGACAGACGAGCAAACCAGAAGCGCTCGAAGGAGAGGTGAGTGTAGGCTGGAGCTGCTTC
icdAredr	GACGTTAAGTCCCCGTTTTTGTTTTTAACAATTATCGTTACATATGAATATCCTCCTTAG
mdhredf	GCAATAGACACTTAGCTAATCATATAATAAGGAGTTTAGGGTGTAGGCTGGAGCTGCTTC
mdhredr	AGAAGCCGGAGCAAAAGCCCCGGCATCGGGCAGGAACAGCCATATGAATATCCTCCTTAG
sucAredf	AGAGTATTAAATAAGCAGAAAAGATGCTTAAGGGATCACGGTGTAGGCTGGAGCTGCTTC
sucBredr	CCTTATCCGGCCTACAGGTAGCAGGTGATGCTCTTGCTGACATATGAATATCCTCCTTAG
sucCredf	GGTCTAAAGATAACGATTACCTGAAGGATGGACAGAACACGTGTAGGCTGGAGCTGCTTC
sucDredr	GAAAACGGACATTTATCTGTTCCCGCAGGAACAGCGAGTTCATATGAATATCCTCCTTAG
sdhCredf	GTCTTAAGGGAATAATAAGAACAGCATGTGGGCGTTATTCGTGTAGGCTGGAGCTGCTTC
sdhBredr	ATAAGACTGTACGTCGCCATCCGGCAACCACTACAACTACCATATGAATATCCTCCTTAG

### Plasmid construction

The *sucCD* genes were PCR amplified from *S*. Typhimurium 4/74 genomic DNA using primers sucF (5′ - TTTTAAGCTTATGAACTTACATGAATATCA) and sucR (5′ - TTTTGGATCCTCCCGCAGGAACAGCGAGTT). The PCR product was digested with BamHI and HindIII, ligated into the low-copy-number vector pWKS30 [25], and transformed into *E. coli* strain DH5α by electroporation [26]. The resulting plasmid was designated pWKS30::*sucCD* and was confirmed by PCR using primers sucF and sucR. Plasmids pWKS30 and pWKS30::*sucCD* were then transformed into 4/74 and AT3449 by electroporation.

### Macrophage infection assays

Infection assays in murine RAW 264.7 macrophages (obtained from American Type Culture Collection; Rockville, MD; ATCC# TIB-71) were performed essentially as previously described [27]. Where indicated, macrophages were activated 24 h prior to infection by seeding the macrophages in MEM culture medium supplemented with 20 units.ml^−1^ (2 ng.ml^−1^) of IFN-γ (Sigma) as previously described [28]. The multiplicity of infection (MOI) for all experiments was 10∶1. The infection assays were allowed to proceed for 2 h and 18 h post infection. To estimate the amount of intracellular bacteria at each time point, cells were lysed using 1% Triton X-100 (Sigma), and samples were taken for viable counts [10]. Statistical significance was assessed by using Student's unpaired *t* test, and a *P* value of 0.05 was considered significant.

### Cytotoxicity assays

Macrophages were infected as described above with the following modifications: DMEM without Phenol Red was used to prevent interference with the assay and the FBS content of the complete tissue culture medium was reduced to 5% due to associated lactate dehydrogenase (LDH) activity. LDH activity released from the mammalian cells was measured in the tissue culture medium using the Cytotox 96 non-radioactive cytotoxicity assay kit (Cat. G1780, Promega) and was considered to reflect cytotoxicity of *S.* Typhimurium. Statistical significance was assessed by using Student's unpaired *t* test, and a *P* value of 0.05 was considered significant.

### HeLa cell infection assays

Infection assays in human HeLa epithelial cells (obtained from American Type Culture Collection; Rockville, MD; ATCC# CCL-2) were performed according to [1]. Approximately, 1×10^5^ HeLa cells were seeded into each well of a 6-well cell culture plates and infected with *S*. Typhimurium 4/74 and mutant strains at an MOI of 10∶1. Prior to infection the *S*. Typhimurium strains had been grown aerobically to an OD_600_ of ∼2.0 in LB broth at 250 rpm at 37°C to allow expression of the SPI-1 Type 3 secretion system.

To increase the uptake of *Salmonella*, the 6-well plates were centrifuged at 1000 g for 5 min, and this was defined as time 0 h. After 1 h of infection, extracellular bacteria were killed with 30 µg.ml^−1^ gentamicin. The media was replaced after 1 h with medium containing 5 µg.ml^−1^ gentamicin. Incubations were continued for 2 h and 6 h post-infection. To determine the levels of intracellular bacteria at each time point, cells were lysed using 0.1% SDS, and samples were taken for viable counts [10]. Statistical significance was assessed by using Student's unpaired *t* test, and a *P* value of 0.05 was considered significant.

### Mouse infection assays

Mouse infection experiments were performed as described [29] with some modifications. Liquid *S.* Typhimurium cultures were grown statically in 50 ml of LB at 37°C overnight in 50 ml Falcon tubes (Corning) [30]. The following day bacteria were resuspended at a final cell density of 1×10^4^ colony forming units (cfu) ml^−1^ in sterile PB*S.* Female BALB/c mice (Charles River U.K. Ltd.) were infected with 200 µl of bacterial suspension via the intraperitoneal (i.p.) cavity at a final dose of 2×10^3^ cfu [29]. The infection was permitted to proceed for 72 h at which time point the mice were sacrificed by cervical dislocation and the spleens and livers surgically removed [29]. Following homogenization of the organs in a stomacher (Seward Tekmar), serial dilutions of the suspensions in PBS were spread onto LB agar plates and bacterial cfu were enumerated after overnight incubation at 37°C [30]. All animal experiments were approved by the local ethics committee and conducted according to guidelines of the Animal Act 1986 (Scientific Procedures) of the United Kingdom.

## Results and Discussion

### Effect of disrupting the TCA cycle on intracellular replication of *S*. Typhimurium in resting macrophages

**Figure 2:**
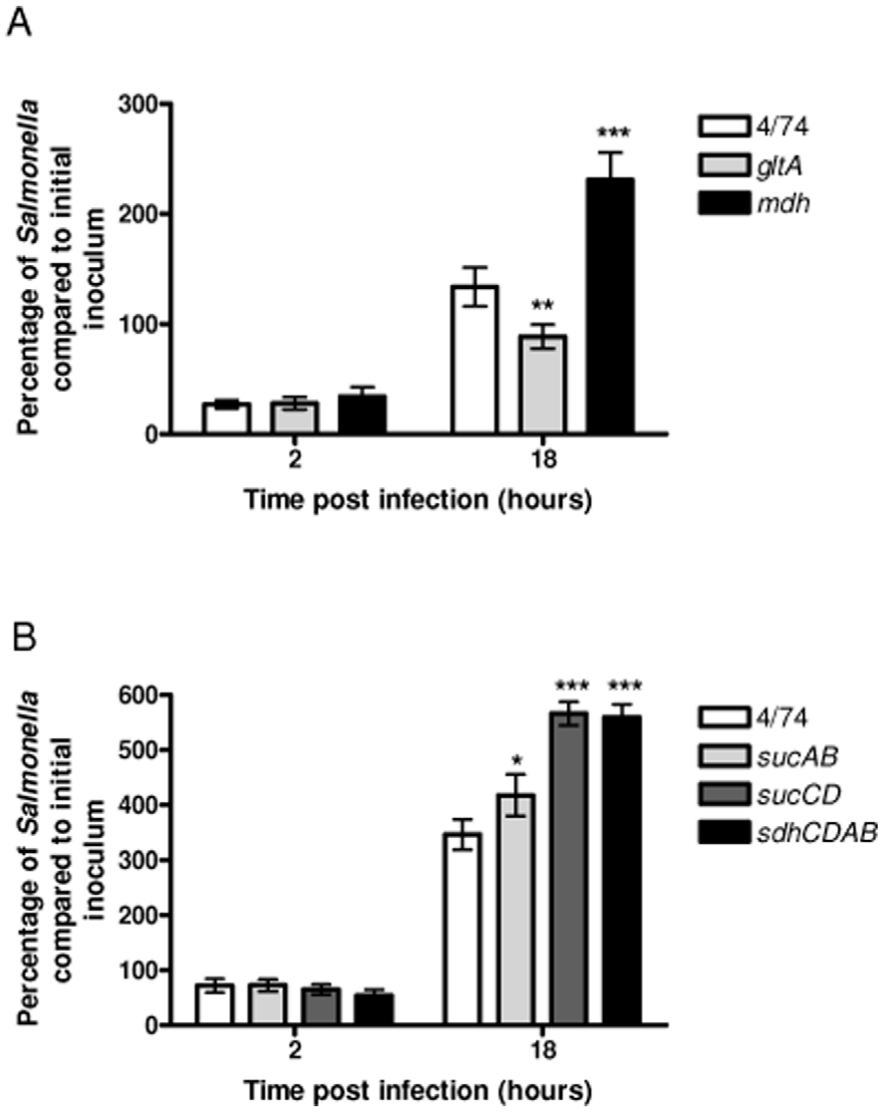
Increased intracellular replication of the *S.* Typhimurium Δ*mdh*, Δ*sucAB*, Δ*sucCD* and Δ*sdhCDAB* strains during infection of resting RAW macrophages. (A) Intracellular replication assays of *S.* Typhimurium 4/74, Δ*gltA* (AT3505) and Δ*mdh* (AT3508) strains during infection of resting RAW macrophages. (B) Intracellular replication assays of *S.* Typhimurium 4/74, Δ*sucAB* (AT3448), Δ*sucCD* (AT3449), and Δ*sdhCDAB* (AT3475) strains during infection of resting RAW macrophages. The data show the number of viable bacteria (expressed as percentages of the initial inocula) within macrophages at 2 h and 18 h post-infection. Each bar represent the statistical mean from three independent biological replicates and the error bars represent the standard deviation (The significant differences between the parental 4/74 strain and the mutant and complemented strains are shown by asterisks *p*>0.05, * *p*<0.05, ** *p*<0.01, and *** *p*<0.001.).

An incomplete TCA cycle has been found in a surprisingly large number of bacterial pathogens including *Helicobacter pylori, Haemophilus influenzae* and *Streptococcus mutans* [31], [32], [33]. The primary role of the TCA cycle is to provide NADH which is used by bacterial cells for ATP synthesis via the electron transport chain (ETC). However, the TCA cycle also plays a key role in the synthesis of intermediates for anabolic pathways; specifically 2-ketoglutarate, oxaloacetate and succinyl-CoA are starting points for the synthesis of glutamate, aspartate and porphyrin respectively (Figure 1). Bacteria that harbor an incomplete TCA cycle retain the ability to generate 2-ketoglutarate, oxaloacetate and succinyl-CoA from pyruvate. To determine the role of the complete TCA cycle for the intracellular replication of *S*. Typhimurium within macrophages we used a genetic approach to interrupt the TCA cycle. We tested *S*. Typhimurium strains carrying deletions of the *gltA, mdh, sdhCDAB*, *sucAB* and *sucCD* genes for their ability to replicate within resting macrophages compared to the wild-type (Figure 2AB). Surprisingly, we recovered up to 40% higher levels of the *S*. Typhimurium Δ*mdh,* Δ*sdhCDAB*, and Δ*sucCD* strains from infected resting macrophages than the wild-type; the level of the *S*. Typhimurium Δ*sucAB* strain was 17% higher than the wild-type (Figure 2B). The apparent ‘over-replication’ phenotypes of the *S*. Typhimurium Δ*mdh*, Δ*sucAB*, Δ*sucCD*, and Δ*sdhCDAB* strains relative to the wild-type suggested that a complete TCA cycle is not necessary for growth within resting macrophages. The observed reduced intracellular replication of the *S*. Typhimurium Δ*gltA* strain compared to the wild-type may suggest that 2-ketoglutarate and therefore glutamate is limiting within the SCV (Figure 2A). This result supports a previous finding that simultaneous prevention of glutamine synthesis and high-affinity transport attenuates *S*. Typhimurium virulence in BALB/c mice and murine macrophages, suggesting a host environment with low levels of glutamine [34].

**Figure 3:**
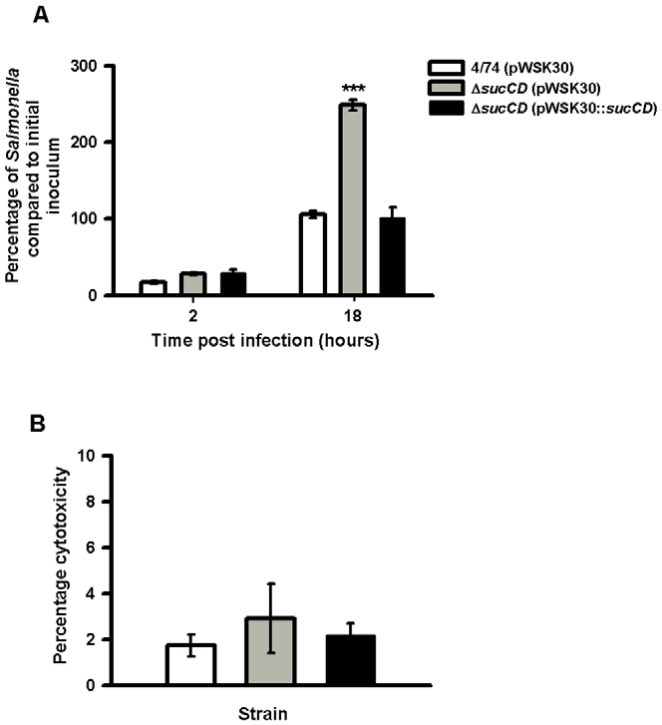
Complementation of the *S*. Typhimurium Δ*sucAB* strain in resting RAW macrophages and cytotoxicity assays. (A) Numbers of viable bacteria (expressed as percentages of the initial inoculum) inside the macrophages at 2 h and 18 h after infection. Each bar indicates the statistical mean for three biological replicates, and the error bars indicate the standard deviations. The significant differences between the parental 4/74 strain and the mutant and complemented strains are shown by asterisks *p*>0.05, * *p*<0.05, ** *p*<0.01, and *** *p*<0.001. (B) Cytotoxicity assays of *S*. Typhimurium wild-type, Δ*sucCD* (AT3449) and complemented Δ*sucCD* (AT???) strains in RAW macrophages after 18 h infection as a percentage of total LDH release from lysed uninfected macrophages. All cytotoxicity data were obtained from three independent biological replicates.

We confirmed that the apparent ‘over-replication’ phenotype of the *S*. Typhimurium Δ*sucCD* strain was due to deletion of the *sucCD* gene. We inserted the *sucCD* gene into the low-copy number vector pWKS30 [25], transformed the construct into the *S*. Typhimurium 4/74 and *S*. Typhimurium strains, and performed infection assays with macrophages. As shown in Figure 3A, the cloned copy of the *sucCD* gene fully complemented the *sucCD* deletion in during infection of macrophages and restored intracellular replication of the *S*. Typhimurium Δ*sucCD* strain containing pWKS30::*sucCD* to wild-type levels. Figure 3B shows that the level of cytotoxicity of the *S*. Typhimurium Δ*sucCD* strain and the complemented strain are indistinguishable from that of the wild-type strain demonstrating that the intracellular replication phenotype of the Δ*sucCD* strain is not a result of increased cell death.

### Increased net replication of the *S*. Typhimurium Δ*sucCD* mutant in resting macrophages is not due to increased flux through the glyoxylate shunt

**Figure 4:**
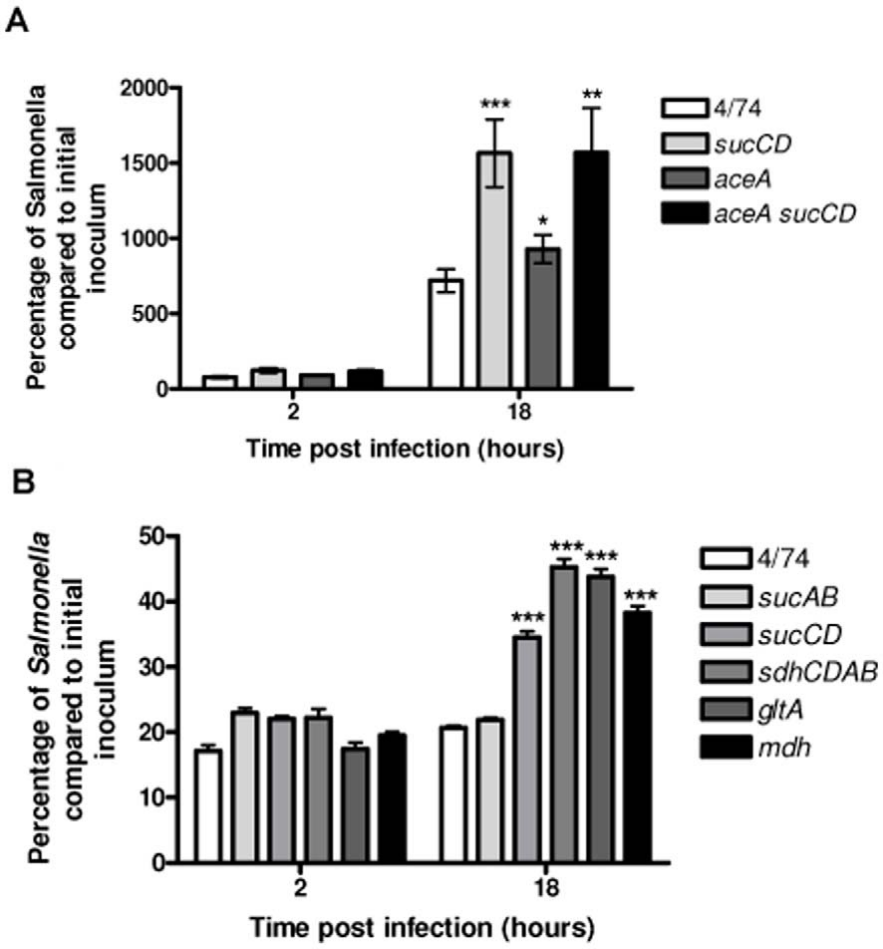
Increased intracellular replication of the *S*. Typhimurium Δ*sucCD*, Δ*sdhCDAB,* Δ*gltA* and Δ*mdh* strains during infection of activated RAW macrophages. (A) Intracellular replication assays of *S.* Typhimurium 4/74, Δ*sucCD* (AT3449), Δ*aceA* (AT3385) and Δ*aceA*Δ*sucCD* (AT3496) strains during infection of resting RAW macrophages (B) Intracellular replication assay of *S.* Typhimurium 4/74, Δ*sucAB* (AT3448), Δ*sucCD*, (AT3449), Δ*sdhCDAB* (AT3475), Δ*gltA* (AT3505) and Δ*mdh* (AT3508) strains during infection of activated RAW macrophages The data show the number of viable bacteria (expressed as percentages of the initial inocula) within activated macrophages at 2 h and 18 h post-infection. Each bar represent the statistical mean from three independent biological replicates and the error bars represent the standard deviation (The significant differences between the parental 4/74 strain and the mutant strains are shown by asterisks *p*>0.05, * *p*<0.05, ** *p*<0.01, and *** *p*<0.001).

The observed increased net replication of the *S*. Typhimurium Δ*mdh,* Δ*sdhCDAB*, Δ*sucAB* and Δ*sucCD* strains could potentially be due to an increased flux through the glyoxylate shunt that conferred a replicative advantage compared to the wild-type within resting macrophages (Figure 2AB). The glyoxylate shunt bypasses reactions of the TCA cycle in which CO_2_ is released, and conserves 4 carbon compounds for biosynthesis (Figure 1) [21]. The pathway is active during growth on 2 carbon compounds such as acetate from fatty acids, when 4-carbon TCA cycle intermediates need to be conserved [21]. The conversion of isocitrate to glyoxylate is catalysed by isocitrate lyase, which has been shown to be required for the persistence of *Mycobacterium tuberculosis* in infected macrophages [21], [35]. Isocitrate lyase is encoded by the *aceA* gene of *S*. Typhimurium [36]. To test whether the glyoxylate shunt plays a role in the increased intracellular replication phenotype of the *S*. Typhimurium Δ*sucCD* strain, we constructed *S*. Typhimurium Δ*aceA* and Δ*sucCD* mutants and tested them for their ability to replicate within resting macrophages. Figure 4A shows that the net intracellular replication of the *S*. Typhimurium Δ*aceA* strain is only slightly increased compared to the wild-type within resting macrophages. Furthermore, combining the Δ*aceA* and Δ*sucCD* mutations does not reduce the increased intracellular replication of the *S*. Typhimurium Δ*sucCD*Δ*aceA* strain to wild-type levels. The data suggest that the glyoxylate shunt does not contribute to the increased net intracellular replication phenotype of the *S*. Typhimurium Δ*sucCD* strain in infected resting macrophages.

### Effect of disrupting the TCA cycle on intracellular replication of *S*. Typhimurium in activated macrophages

In the light of the above results, one possibility is that the net increase in intracellular replication of the *S*. Typhimurium Δ*mdh*, Δ*sucAB*, Δ*sucCD*, and Δ*sdhCDAB* strains may be due to their enhanced ability to survive the antimicrobial defense mechanisms of macrophages, which include the ROI and RNI response [37], [38]. Production of ROI and RNI species are stimulated during activation of macrophages by lipopolysaccharide and IFN-γ [39]. We therefore tested the ability of *S*. Typhimurium Δ*sucAB*, Δ*sdhCDAB,* Δ*mdh*, Δ*sucCD* and Δ*gltA* mutants to replicate within activated macrophages compared to the wild-type (Figure 4B). In this experiment, the wild-type 4/74 strain showed no net replication within activated macrophages; however the Δ*sdhCDAB,* Δ*mdh*, Δ*sucCD* and Δ*gltA* strains all showed increased net replication (Figure 4B). The observation that the *S*. Typhimurium Δ*sucAB* shows no net replication within activated macrophages may reflect limited availability of succinyl-CoA within the SCV of activated macrophages. Succinyl-CoA is required for the biosynthesis of lysine, methionine and diaminopimelate. Diaminopimelate is essential for the synthesis of peptidoglycan and hence the bacterial cell wall [40], [41]. *S*. Typhimurium SR11 Δ*sucAB* strains have been shown to be avirulent in BALB/c mice [17].

The increased levels of the *S*. Typhimurium Δ*gltA* strain within activated macrophages, compared to decreased intracellular replication in resting macrophages may reflect increased survival rather than growth of the *S*. Typhimurium Δ*gltA* strain within activated macrophages. The proposed ability of the *S*. Typhimurium Δ*sdhCDAB,* Δ*mdh*, Δ*sucCD* and Δ*gltA* mutants to better survive the antimicrobial ROI and RNI response in macrophages compared to the wild-type strain prompted us to test an alternate cell line (HeLa epithelial cells) in which *S*. Typhimurium is still within a vacuole, but not subject to the ROI and RNI responses [1].

### Effect of disrupting the TCA cycle on intracellular replication of *S*. Typhimurium in epithelial cells

**Figure 5:**
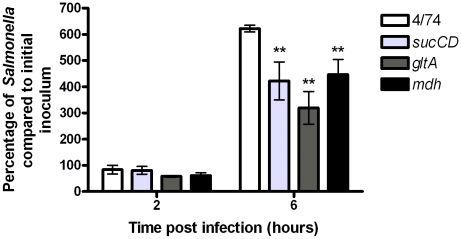
Decreased intracellular replication of *S*. Typhimurium Δ*sucCD* and Δ*gltA* strains during infection of HeLa epithelial cells. Intracellular replication assays of *S.* Typhimurium 4/74, Δ*sucCD* (AT3449), Δ*gltA* (AT3505) and Δ*mdh* (AT3508) strains during infection of HeLa cells. The data show the number of viable bacteria (expressed as percentages of the initial inocula) within macrophages at 2 h and 6 h post-infection. Since *S*. Typhimurium initiates intracellular replication much earlier in HeLa cells (3–4 h) compared to macrophages (∼8 h post-infection) replication was assessed at 6 h post-infection (Hautefort *et al*., 2008). Each bar represent the statistical mean from three independent biological replicates and the error bars represent the standard deviation (The significant differences between the parental 4/74 strain and the mutant strains are shown by asterisks *p*>0.05, * *p*<0.05, ** *p*<0.01, and *** *p*<0.001).

Figure 5 shows that the *S*. Typhimurium Δ*sucCD* and *ΔgltA* strains show 30% less intracellular replication than the wild-type strain between 2 h and 6 h post infection. The *S*. Typhimurium Δ*mdh* strain showed the same level as the wild-type.

These observations suggest that, in contrast to resting and activated macrophages, an intact TCA cycle is required for optimal intracellular replication within epithelial cells. Since HeLa cells lack the antimicrobial ROI and RNI responses deployed by macrophages, the reduced intracellular replication of the *S*. Typhimurium Δ*sucCD* and Δ*gltA* strains in epithelial cells supports the possibility that a disrupted TCA cycle favors the ability of *S*. Typhimurium to survive macrophage defense mechanisms [1]. Disruption of the TCA cycle may result in decreased flux through the ETC which could enhance the ability of the Δ*sdhCDAB,* Δ*mdh*, Δ*sucCD* and Δ*gltA* mutants to survive the antimicrobial response mounted by activated macrophages by reducing Fenton reaction-based oxidative damage [38]; this hypothesis is the subject of further investigation, although we cannot rule out the possibility that other differences between the mutant and wild-type strains contribute to their infection phenotypes at this stage. For example, disruption of the TCA cycle may alter the redox balance in *Salmonella* to reduce endogenous NADH levels and therefore ETC activity. This would reduce endogenous Fenton-based oxidative damage and may enhance intracellular survival. Interestingly it has been postulated that bactericidal antibiotics may act by stimulating the TCA cycle which would increase NADH flux through the ETC thus increasing hydroxyl radical formation via the Fenton reaction and causing cell death [42], [43].

### Effect of disrupting the TCA cycle on infection of mice by *S*. Typhimurium

The increased net replication of the *S*. Typhimurium Δ*sdhCDAB,* Δ*mdh*, Δ*sucCD* and Δ*gltA* strains in macrophages was surprising in the light of previous findings which showed that *S*. Typhimurium Δ*sucAB*, Δ*sdhCDAB* and Δ*sucCD* mutants were attenuated in orally infected mice [17]. We therefore tested our *S*. Typhimurium Δ*sucAB* and Δ*sucCD* strains for their ability to infect mice.

**Figure 6:**
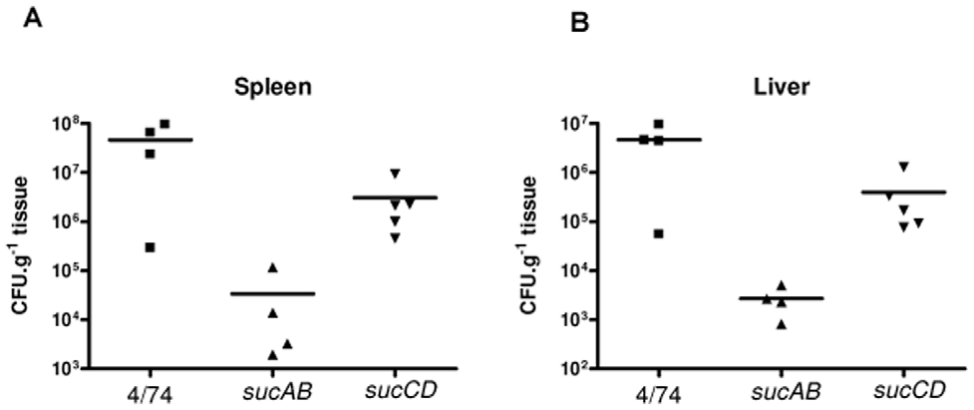
Succinyl-CoA synthetase and α-ketoglutarate synthetase are required for successful infection of mice. Colony forming units per gram of tissue recovered from the spleens and livers of BALB/c mice following i.p. infection for 72 h with *S.* Typhimurium 4/74, Δ*sucAB* (AT3448) and Δ*sucCD* (AT3449).

Following infection of mice, *S*. Typhimurium disseminates systemically from the Peyer's patches to the liver and spleen where it continues to grow within macrophages [44], [45], [46]. We inoculated mice with the *S*. Typhimurium Δ*sucAB*, Δ*sucCD* and wild-type strains via the intraperitoneal route. *Salmonella* bacteria were recovered and enumerated after 72 h of infection. The results demonstrate that the *S*. Typhimurium Δ*sucAB* mutant is severely attenuated (by ∼10^3^ –fold) compared to the wild-type in the spleens and livers of infected mice (Figure 6A). The *S*. Typhimurium Δ*sucCD* strain is less attenuated than the *S*. Typhimurium Δ*sucAB* strain in livers and spleens of infected mice, but is still reduced by ∼10 –fold compared to the wild-type (Figure 6B). These results are consistent with the finding of Tchawa Yimga *et al*., [17], who found that *S*. Typhimurium SR11 Δ*sucAB* was avirulent and the Δ*sucCD* mutant was attenuated in orally infected BALB/c mice. The contrast between the attenuation of the *S*. Typhimurium Δ*sucAB* and Δ*sucCD* mutants in mice, and their increased intracellular replication in resting and activated macrophages suggest that *Salmonella* may encounter environments within the host where a complete TCA cycle is advantageous.

### Summary

We investigated the ability of *S*. Typhimurium TCA cycle mutants to replicate within macrophages, HeLa epithelial cells, and to infect BALB/c mice. The *S*. Typhimurium Δ*mdh*, Δ*sucCD*, Δ*sdhCDAB* strains replicated to higher levels than the wild-type within resting and activated macrophages, suggesting an enhanced ability to survive antimicrobial mechanisms. This is supported by the observed decreased levels of the *S*. Typhimurium Δ*sucCD* and Δ*gltA* strains within infected epithelial cells, which lack an ROI and RNI response, although we cannot rule out other mechanisms at this stage. The increased level of the *S*. Typhimurium Δ*sucCD* in resting macrophages was not due to increased flux through the glyoxylate shunt. In agreement with previous work, we found that the S. Typhimurium Δ*sucAB* and Δ*sucCD* strains are attenuated in mice, suggesting an intact TCA cycle is required for successful infection within specific host environments.

## Supporting Information

Table S1 Strains and plasmids used in this study.
(0.03 MB PDF)
