# Supplementary material for: An Incomplete TCA Cycle Increases Survival of Salmonella Typhimurium during Infection of Resting and Activated Murine Macrophages
Source: PLoS One. 2010 Nov 8;5(11):e13871. doi: 10.1371/journal.pone.0013871 (PMC2975626; doi:10.1371/journal.pone.0013871)
Supplement: Table S1 — Strains and plasmids used in this study. (0.03 MB PDF) [file pone.0013871.s001.pdf]

Table S1. Strains and plasmids used in this study.

S. **Typhimurium strains**	**Relevant genotype**	**Method of construction**	**Reference**
4/74	Wild-type strain	N/A	[22]
AT3385	4/74 Δ*aceA*::Cm	λ Red mutagenesis*: pKD3 PCR product using primers aceAredf and aceAredr	This study
AT3448	4/74 Δ*sucAB*::Kn	λ Red mutagenesis*: pKD4 PCR product using primers sucAredf and sucBredr	*ibid*
AT3449	4/74 Δ*sucCD*::Kn	λ Red mutagenesis*: pKD4 PCR product using primers sucCredf and sucDredr	*ibid*
AT3462	4/74 Δ*sdhCDAB*::Kn	λ Red mutagenesis*: pKD4 PCR product using primers sdhCredf and sdhBredr	*ibid*
AT3469	4/74 Δ*aceA*	Transformed JH3385 with pCP20 to excise the Cm^R cassette from the chromosome. The plasmid was cured from the strain.	*ibid*
AT3496	4/74 Δ*sucCD*::Kn, Δ*aceA*	P22 transduced Δ*sucCD*::Kn from JH3449 into JH3469	*ibid*
AT3472	4/74 Δ*sucAB*	Transformed JH3448 with pCP20 to excise the Kn^R cassette from the chromosome. The plasmid was cured from the strain.	*ibid*

AT3475	4/74 Δ*sdhCDAB*	Transformed JH3462 with pCP20 to excise the Kn^{R} cassette from the chromosome. The plasmid was cured from the strain.	*ibid*
AT3477	4/74 Δ*sucCD*	Transformed JH3449 with pCP20 to excise the Kn^{R} cassette from the chromosome. The plasmid was cured from the strain.	*ibid*
AT3505	4/74 Δ*gltA*::Kn	λ Red mutagenesis*: pKD4 PCR product using primers gltAredf and gltAredr	*ibid*
AT3508	4/74 Δ*mdh*::Kn	λ Red mutagenesis*: pKD4 PCR product using primers mdhredf and mdhredr	*ibid*
**Plasmids**			
pKD46	λ Red recombinase expression plasmid	N/A	[23]
pKD3	Cm^{R} resistance cassette-containing plasmid	N/A	*ibid*
pKD4	Kn^{R} resistance cassette-containing plasmid	N/A	*ibid*
pCP20	FLP-recombinase expression plasmid	N/A	*ibid*
pWKS30	Ap^{R} low-copy-number vector,<br>pSC101 origin of replication	N/A	[25]
pWKS30::*sucCD*	Ap^{R} low-copy-number vector, pSC101 origin of replication, expresses *sucCD*	2,056 bp sucF-sucR PCR product cloned into pWKS30 BamHI-HindIII sites	This study

*The λRed mutagenesis method is described in Materials and Methods
